# One-Stage Formation of Two-Dimensional Photonic Crystal and Spatially Ordered Arrays of Self-Assembled Ge(Si) Nanoislandson Pit-Patterned Silicon-On-Insulator Substrate

**DOI:** 10.3390/nano11040909

**Published:** 2021-04-02

**Authors:** Alexey V. Novikov, Zhanna V. Smagina, Margarita V. Stepikhova, Vladimir A. Zinovyev, Sergey A. Rudin, Sergey A. Dyakov, Ekaterina E. Rodyakina, Alexey V. Nenashev, Sergey M. Sergeev, Artem V. Peretokin, Anatoly V. Dvurechenskii

**Affiliations:** 1 Institute for Physics of Microstructures Russian Academy of Sciences, 603950 Nizhny Novgorod, Russia; mst@ipmras.ru (M.V.S.); sj@ipmras.ru (S.M.S.); aperetokin@ipmras.ru (A.V.P.); 2 Radiophysical Department, Nizhny Novgorod State University, 603950 Nizhny Novgorod, Russia; 3 Rzhanov Institute of Semiconductor Physics, Siberian Branch of Russian Academy of Sciences, 630090 Novosibirsk, Russia; smagina_isp@mail.ru (Z.V.S.); zinoviev19@mail.ru (V.A.Z.); rudin@isp.nsc.ru (S.A.R.); rodyakina@isp.nsc.ru (E.E.R.); nenashev@isp.nsc.ru (A.V.N.); dvurech@isp.nsc.ru (A.V.D.); 4 Center for Photonics and Quantum Materials, Skolkovo Institute of Science and Technology, Nobel Street 3, 143026 Moscow, Russia; s.dyakov@skoltech.ru; 5 Physical Department, Novosibirsk State University, 630090 Novosibirsk, Russia; 6 Institute of Nuclear Power Engineering and Applied Physics, Alekseev State Technical University of Nizhny Novgorod, 603155 Nizhny Novgorod, Russia

**Keywords:** Ge(Si) islands, quantum dots, pre-patterned substrate, Monte-Carlo simulation, spatial ordering, luminescence, photonic crystal

## Abstract

A new approach to improve the light-emitting efficiency of Ge(Si) quantum dots (QDs) by the formation of an ordered array of QDs on a pit-patterned silicon-on-insulator (SOI) substrate is presented. This approach makes it possible to use the same pre-patterned substrate both for the growth of spatially ordered QDs and for the formation of photonic crystal (PhC) in which QDs are embedded. The periodic array of deep pits on the SOI substrate simultaneously serves as a template for spatially ordering of QDs and the basis for two-dimensional PhCs. As a result of theoretical and experimental studies, the main regularities of the QD nucleation on the pre-patterned surface with deep pits were revealed. The parameters of the pit-patterned substrate (the period of the location of the pits, the pit shape, and depth) providing a significant increase of the QD luminescence intensity due to the effective interaction of QD emission with the PhC modes are found.

## 1. Introduction

The application of optical resonators is one of the physical approaches widely used to improve the efficiency of light-emitting media [1,2,3,4,5,6]. Among a wide range of semiconductor structures, the problem of increasing the quantum efficiency is particularly relevant for light-emitting SiGe heterostructures due to a number of reasons. First, the efficiency of radiative recombination in these structures is relatively low, which is a consequence of the indirect bandgaps of both Si and Ge. Second, among the variety of materials offered to solve the problem of the lack of an effective light source for silicon nanophotonics and optoelectronics, SiGe heterostructures are the most compatible with modern CMOS technology [7,8,9].

Among the wide class of SiGe heterostructures, the structures with the self-assembled Ge(Si) nanoislands and quantum dots (hereinafter referred to as QDs) seem to be the most attractive for the application in resonators. The spatial localization of charge carriers in nanoislands leads to the low sensitivity of their luminescent properties to various structural defects [10,11], including the developed surface of many resonators. The emission of Ge(Si) QDs is observed in the practically important wavelength range of 1.3–1.55 µm [12,13,14]. An additional advantage of Ge(Si) QDs is the possibility to grow them directly on the silicon-on-insulator (SOI) substrates without a thick intermediate buffer [8]. This makes it easy to implementlight confinement in a growth direction due to the total internal reflection, which greatly facilitates the formation of resonators.

The efficiency of Ge(Si) QDs interaction with cavity modes can be significantly improved due to the precise positioning of QDs inside the cavity as demonstrated in [15,16] for two-dimensional photonic crystals (PhCs) with micro-resonators (micro-cavities). One should note the growing interest in the interaction of the light-emitting media not only with the cavity modes but also with the modes of photonic crystals, including the so-called bound states in the continuum (BIC) standing out by their high Q factors [17,18]. To ensure the effective interaction of Ge(Si) QDs with these modes, it is important to realize a spatial ordering of a large number of Ge(Si) QDs in the entire photonic crystal [19,20]. The standard approach, in this case, is a two-stage process, which includes: (1)—the formation of an ordered Ge(Si) nanoisland array on a silicon substrate, and (2)—the formation of the photonic crystal itself with the precise positioning of holes relative to Ge(Si) nanoislands [19,20]. This approach and the methods for the spatial ordering of single QDs inside the pits on the pre-patterned substrates are well developed (see reviews [8,21,22]).

Meanwhile, recent studies show that in some cases, the stages of spatial ordering of Ge(Si) nanoislands and PhC formation can be combined [23]. For that, it is necessary to provide the nucleation of Ge(Si) QDs not inside the pits on the pre-patterned substrate but between them or along the perimeter of pits. In this case, the arrays of pits should form photonic crystals with the modes which fall into the spectral range of the luminescence signal of Ge(Si) QDs.

In this paper, we present the study of the nucleation processes of Ge(Si) QDs on pit-patterned Si substrates by Monte-Carlo (MC) simulation. This study made it possible to choose the parameters of the pit arrays providing the nucleation of QDs between or around the pits rather than inside them. Making use of these results, the structures with Ge(Si) QD groups arranged around the pits on SOI substrate were obtained. The significant increase of the luminescence response associated with Ge(Si) QDs was observed at certain periods of pits arrangement, which is attributed to the interaction of QDs emission with the modes of PhC formed by the pits.

## 2. Materials and Methods

To elucidate the main mechanisms of the Ge QDs nucleation on a pit-patterned Si(001) surface, the growth processes were simulated by the MC method using the previously developed three-dimensional (3D) kinetic model of heteroepitaxial growth [24]. The model is based on a 3D diamond-like crystal lattice, each lattice node of which could be occupied by either the Si or Ge atoms or could be empty.

To account for the elastic interaction between atoms, a displacement vector is assigned to each atom. The displacement vector gives a relative displacement with respect to the radius vector of an ideal crystal lattice site. Epitaxial growth is modeled as a sequence of elementary events randomly chosen in accordance with their probabilities. There are three types of events: the addition of a new atom (deposition), diffusion jump of the deposited atom on the surface, and a random displacement of an atom around its equilibrium position.

The deposition probability is determined according to the required growth rate.The probability of a diffusion jump is defined according to the following two conditions: (i) the probability of a jump depends only on the positions of the neighbors of the jumping atom within the second coordination sphere; (ii) the probabilities of forwarding and backward jumps must satisfy the principle of detailed balance. The activation energy of the diffusion jumps in the model contains a negative term proportional to the number of interatomic bonds and the number of pairs of next-to-nearest neighbors, and a positive term equal to the elastic energy expressed in the form of the Keating potential [25]. The random displacements obey the Boltzmann distribution.

In the model, the shape of the pits and their relative sizes were scaled down by the factor of 35 with respect to the experimental data. Periodical boundary conditions were used at the lateral sides of simulated samples, which correspond to the case of an infinite periodic array of pits arranged in a square lattice. The slab surface was (001)-oriented. The simulated Ge deposition was carried out at the rate of 0.1 ML/s (1 monolayer (ML) ~ 0.14 nm) and a temperature of 450 °C.

The formation of arrays of spatially ordered Ge(Si) QDs was carried out by the molecular beam epitaxy on SOI substrates using Riber SIVA-21 MBE system. SOI substrates with a buried SiO_2_ layer thickness of 3 µm and a top Si layer thickness of 80–90 nm were used. Before the growth, a periodic array of round-shaped pits was created using electron beam lithography and plasma chemical etching. The pits were located in the nodes of a square lattice with a period from 0.5 to 2 µm. The shape and diameter of the pits depended on the electron beam lithography exposure time and the etching conditions. The depth of the pits was determined by the etching time. The MC growth model results were taken into account in determining the parameters of the pit arrays.

Growth on the pre-patterned SOI substrates began with the deposition of a Si buffer layer of 50–100 nm thick. Then 4 ML of Ge weredeposited at 700 °C, which is sufficient for the formation of Ge(Si) QDs at the pit-patterned region of the substrate [26]. The Ge deposition rate was 0.05 Å/s. The multilayer structure containing four layers of ordered Ge(Si) QDs was formed for luminescent measurements. The thickness of the Si spacers between the QD layers was 15 nm, whichenables the vertical alignment of QDs [27]. To control the spatial arrangement of QDs, the growth sequence was completed by the formation of the upper layer of Ge(Si) uncapped QDs. Details of the structure growth can be found in [23,26].

The morphology of pit-patterned substrates and grown structures with spatially ordered Ge(Si) QDs was studied using atomic force microscopy (AFM) (Solver P47-PRO, NT-MDT Spectrum Instruments, Moscow, Russia) and scanning electron microscopy (SEM) (Hitachi SU8220, Hitachi, Tokyo, Japan), including the use of focused ion beam etching (FIB) to analyze the cross-section of the structure.

The luminescent properties of fabricated structures were studied using the micro-photoluminescence (micro-PL) with high spatial and spectral resolution. The micro-PL signal was excited by a continuous laser at a wavelength of 532 nm. The measurements were carried out in the normal incidence geometry of both the exciting laser beam and the detected PL signal. A Mitutoyo M Plan APO NIR 10× (NA = 0.26) (Mitutoyo, Kawasaki, Japan) was used for the focusing of a laser beam on the sample surface of aspot with a diameter of ~10 µm. The high spectral resolution (4 cm^−1^) in the experiment was provided by a Fourier spectrometer Bruker IFS 125 HR (Bruker, Berlin, Germany). The luminescence signal was detected by a liquid nitrogen-cooled Ge detector. All measurements were carried out at a temperature of 77 K.

The optical properties of the studied two-dimensional PhCs and their mode structure were simulated using the Fourier modal method in the scattering matrix form [28], also known as rigorous coupled-wave analysis (RCWA) [29].

## 3. Results and Discussion

### 3.1. MC Simulation of Heteroepitaxial Growth of Ge on a Pit-Patterned Si(001) Surface

Numerical simulation of QD growth on a structured Si(001) surface allows us to reveal the main features of the QD nucleation on the pit-patterned substrates, in particular, the dependences on the pit shape, the period of their location, and the depth ofpits. A study of QD growth depending on the pit shape demonstrates that in the case of V-shaped pits, the QD nucleation occurs inside the pits. Whereas, in the case of U-shaped pits (with a flat bottom), QDs nucleate at their periphery [30]. This effect is a consequence of the different strain distribution at the Ge/Si interface at the bottom of U- and V-shaped pits (Figure S1 in Supplementary Materials). The most relaxed region (more favorable for nucleation) for a V-shaped pit is located in the center of the pit, while for a U-shaped pit the most relaxed regions are located at the boundary between the flat bottom and the pit walls. For more details of the nucleation dependence on the pit shape, see Supplementary Materials.

With an increase in the amount of deposited material for V-shaped pits, the nucleation also occurs at the periphery of pits (near the outer boundary of pits). Here the additional nucleation sites become effective at the certain inclination angles of the pit sidewalls (>30°) due to elastic relaxation in the convex areas of the pre-patterned substrate [31]. Starting from a certain moment, the nucleation of Ge QDs becomes energetically more favorable at the edges of the pits than inside them. In contrast, for U-shaped pits, there areno principal changes in the nucleation pattern with increasing the amount of deposited Ge; the QDs nucleate only at their periphery.

**Figure 1:**
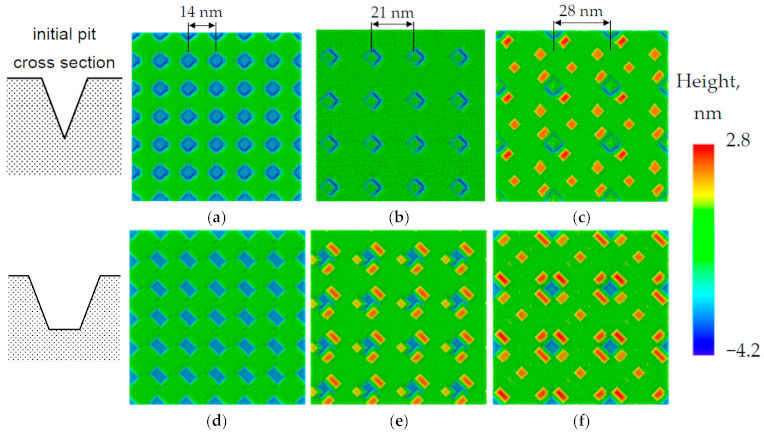
Calculated morphology of the pit-patterned Si(001) substrate with V-shaped (**a**–**c**) and U-shaped (**d**–**f**) pits after deposition of 5 ML of Ge. The initial cross-section for V- and U-shaped pits is presented to the left of corresponding calculated results. The growth temperature is 450 °C. The rate of Ge deposition is 0.1 ML/s. The distance between the pits is 14 nm (**a**,**d**), 21 nm (**b**,**e**) and 28 nm (**c**,**f**). For all figures, the diameter of pits is 13.6 nm, their depth is 2.8 nm, and the pit sidewall inclination angle is 45°. All sizes are scaled down by the factor of 35 with respect to the experimental data. The zero height level in the color legend corresponds to the flat surface level between the pits.

The MC simulation of Ge/Si heteroepitaxy on the substrates with different period of a square lattice of pits demonstrates that the increase in the lattice period leads to an increase in the size of Ge QDs or their number in the area surrounding each pit (at the same amount of deposited Ge). To understand this result, one should keep in mind that in our growth conditions, the adatom diffusion length exceeds the distance between pits. Therefore all adatoms can reach the pits and participate in the nucleation of QDs near (or inside) the pits. The larger the lattice period, the more material can be accumulated by each pit. For example, QDs do not nucleate on the surface with lattice period d = 14 nm, whereas, for the larger period, d = 21 nm, the nucleation of islands occurs inside the pits (Figure 1a,b). For the structures with an even larger period (28 nm) the nucleation of QDs occurs both inside and outside the pits (Figure 1c). In the case of U-shaped pits, we obtain similar results, but the nucleation of QDs occurs only near the pits, but not inside them (Figure 1d–f). In both of the presented cases, the number of QDs nucleated around the pits increases with the distance between pits.

**Figure 2:**
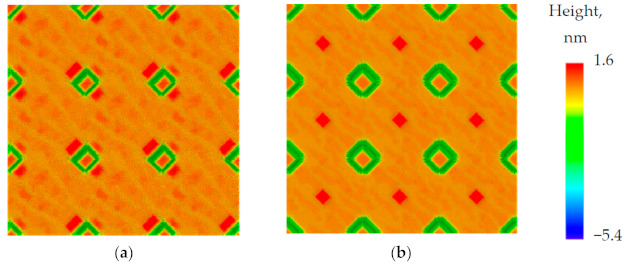
Calculated morphology of the pit-patterned Si(001) substrate with V-shaped pits with different inclination angles of sidewalls (**a**) α = 30° and (**b**) α = 54° after deposition of 4 ML and 5 ML of Ge, respectively. The growth temperature is 450 °C. The rate of Ge deposition is 0.1 ML/s. The zero height level in the color legend corresponds to the flat surface level between pits.

The modeling results for QD nucleation depending on the pit depth are shown in Figure 2. The dependence on the pit depth can be reduced to the dependence on the inclination angle of pit side walls [8,32]. For shallow V-shaped pits (inclination angle 30°–45°), QDs nucleate first inside the pits and then (at a larger amount of deposited material) around the pits near their edges (Figure 2a). In the case of deeper pits (inclination angle of pit sidewalls > 54°), one large QD is initially formed inside the pit, and then, as the amount of deposited Ge increases, smaller QDs are nucleated between the pits at the maximum distance from their edges, just exactly in the middle between the pits (Figure 2b). To understand this result, one should take into account that during the growth, the pits are faceted by {111} planes with a high adatom diffusion coefficient. The adatoms captured by the pit walls instantly reach the center of the pit and are captured by the island. In this case, the pit can be considered as an almost ideal sink for adatoms, and the adatom concentration at the pit edge is not enough for the island nucleation.

In the case of U-shaped pits, the QD nucleation always takes place around the pits, regardless of the inclination angle of the sidewalls and the depth of the pits.

**Figure 3:**
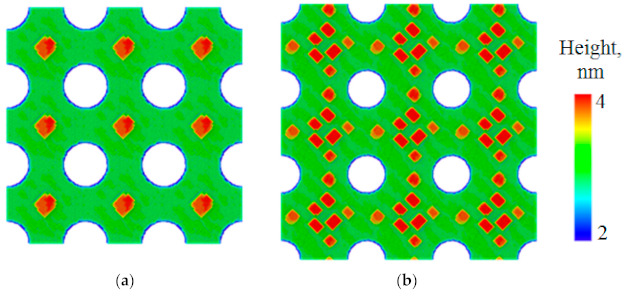
Simulated arrangement of 3D Ge islands nucleated in periodic structure with infinitely deep pits depending on the distance between them. The distance between the pits is 24.5 nm (**a**) and 31.5 nm (**b**). The growth temperature is 450 °C. The rate of Ge deposition is 0.1 ML/s. The amount of Ge was 10 ML for (**a**) and 6 ML for (**b**). The pit diameter is 13.6 nm. All sizes are scaled down by the factor of 35 with respect to the experimental data.

The MC simulation was also carried out for “infinitely” deep pits, where atoms trapped inside the pit (at the pit bottom) were removed from the simulated system, i.e., the pits servedas ideal sinks for adatoms. This simulation shows that at a certain distance between pits, the QD formation occurs at the maximum possible distance from the pits (exactly in the middle between the pits) (Figure 3a), as well as for the finite-depth pits with the V-shaped bottom, having an inclination angle of the sidewalls of 54°. The number of islands surrounding the “infinitely” deep pits increases with the distance between pits (Figure 3b).

The results of MC simulation of Ge heteroepitaxial growth on pit-pattered Si(001) substrates have shown that by choosing the structured surface parameters (the pit period, shape, and depth), various conditions for the ordered QD growth can be realized. To solve the problem of the simultaneous formation of both a two-dimensional PhC and an array of ordered Ge QDs, it is necessary to create pits with large (>54°) inclination angles of the side walls or U-shaped pits. It enables us to realize the nucleation of QDs either around the pits (near their edges) or at the maximum distance from the pits (in the middle between adjacent pits). It opens the possibility of fabricating an optimized silicon pit-pattered surface where the spatial position of Ge QDs would correspond to the local maxima of the electromagnetic field. As has been shown in [33], by placing QDs to a proper position within the emitting structure, one can achieve a significantly higher external quantum yield compared to a case of a uniform spatial distribution of QDs.

### 3.2. Formation of an Array of Spatially Ordered Ge(Si) QDs

Experimental studies of Ge growth on a pit-patterned Si(001) surface are in good agreement with the simulation results. Recently it was shown that for the same period of pits arrangement in a square lattice, the nucleation of Ge(Si) QDs occurs at the bottoms of the V-shaped pits and the periphery of the U-shaped pits [34]. For structures with shallow pits (15–20 nm deep) with the sidewall inclination angle ˂30°, islands nucleate inside the pits [26]. In the structures with the depth of the pits more than 50 nm (the inclination angle larger than 54°), islands are formed only at the periphery of the pits. At intermediate depths of the pits (in the range of the pit’s depth 20–50 nm), islands nucleate both inside the pits and around them.

In this work, for the single-stage formation of a two-dimensional photonic crystal and an ordered array of Ge(Si) QDs, we investigated the growth of Ge on the patterned SOI substrates with a square-lattice arrangement of the pits, with a sidewall inclination angle significantly greater than 54°. The pit diameter was 200 nm before the structure growth. The pit period in a square lattice was varied from 0.5 to 2 µm with a step of 0.1 µm. The choice of the range of periods was determined by the fact that the most significant changes in the mechanism of Ge(Si) island nucleation on the pit-patterned Si(001) surface occur in this range [20,26]. Also, according to calculations, PhC modes characterized by different Q-factor values for these periods of the photonic crystal are fall in the energy range of 0.8–1.05 eV corresponding to the spectral position of the PL signals from Ge(Si) QDs and the wetting layer.

**Figure 4:**
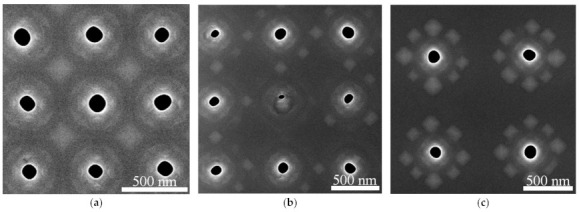
SEM images of ordered arrays of QDs produced by the deposition of ~4 ML of Ge on patterned SOI substrates with pit periods of 0.5 µm (**a**), 0.7 µm (**b**), 1 µm (**c**). The dark areas correspond to pits; the bright areas correspond to Ge(Si) islands and elevations on the growth surface. The deposition temperature is 700 °C. The rate of Ge deposition is 0.05 Å/s.

The results of AFM and SEM studies of structures with small (0.5–0.6 µm) lattice periods are in qualitative agreement with the simulation results (Figure 3a). In the specified range of periods, the QDs nucleation starts at the maximum distance from the pits, that is, in the centers of the squares formed by neighboring pits (Figure 4a). It should be noted that the amount of Ge atoms per pit at the lattice period 0.5–0.6 µm is apparently insufficient for the formation of well-defined faceted three-dimensional islands [26]. Instead of this, the Ge growth on the pit-patterned surface is characterized by the formation of shallow unfaceted mounds, which are located equidistantly from the pits (Figure 4a). This corresponds to the initial stage of the barrierless formation of 3D Ge(Si) islands, which is usually observed at sufficiently high growth temperatures (≥600 °C) [35,36].

**Figure 5:**
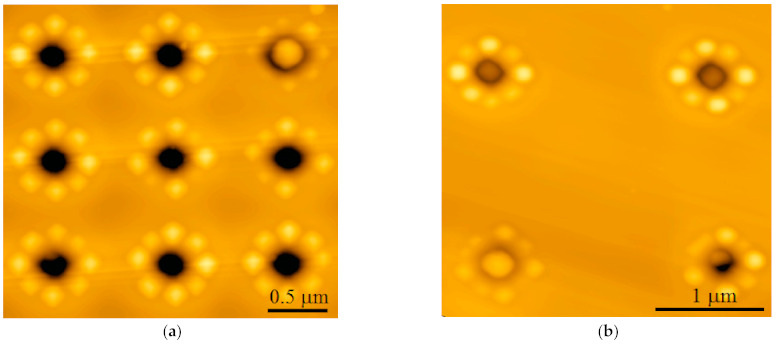
AFM images of the ordered arrays of QDs formed after the deposition of ~4 ML of Ge on patterned SOI substrates with pit periods of 1 µm (**a**) and 2 µm (**b**). The color scale of heights is selected in such a way as to highlight the QDs against the pits.

At the used amount of deposited Ge the formation of pyramidal Ge(Si) QDs with {105} facets is observed with an increase of the lattice period to 0.7 µm (Figure 4b). For this period, the amount of Ge atoms per pit is sufficient for the formation of several islands at a considerable distance from the edges of the pits (Figure 4b). An increase of the pit period to 1 µm leads to the increase of the average number of islands forming around the pit up to 8–10 (Figure 4c and Figure 5a). In this case, the interaction between the pits can be neglected in accordance with the simulation results. The islands are grouped exclusively around the pits.

The increase of the lattice period to 2 µm does not change the island arrangement qualitatively, as compared to the period of 1 µm. At the same time, the number of islands grouped around the pits stabilizes at the level of 8, and their average size increases (Figure 5). The latter circumstance is associated with an increase of the amount of deposited Ge atoms per pit with the increase of pit period.

**Figure 6:**
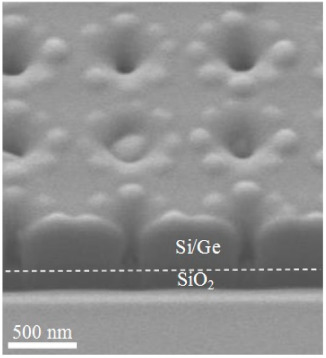
SEM image of a structure cross-section for pit period of 0.8 µm. The dotted line is drawn for clarity and separates the region of the buried SiO_2_ layer and the top Si+epitaxially grown layers.

According to SEM and AFM studies, at lattice periods ≤1 µm, the majority of pits remains to be not overgrown after the epitaxy (Figure 4 and Figure 5). Analysis of the SEM images of samplecross-sectionsobtained by FIB (Figure 6) showed that for such periods, thepit depth coincides with the thickness of the entire structure above the buried oxide, which is ~260 nm. The diameter of the pits is smaller at the surface of the structure than at its bottom (Figure 6). This could be explained by the partial overgrowth of pits during the material deposition leading to the gradual decrease of their diameter. The number of overgrown pits increases significantly with an increase of pits period from 1 to 2 microns (Figure 5), which is due to an increase in the amount of deposited material (Si and Ge) per pit. When the pit is overgrown, the material accumulates in its center, and large Ge(Si) island is formed (see Figure 5 and Figure 6).

The growth of ordered Ge(Si) QDs on a patterned surface of SOI substrate with pits having large (more than 60°) sidewall inclination angles, depending on the pit period, qualitatively coincides with the simulation results for the case of “infinitely deep” pits. At a growth temperature of 700 °C and a fixed thickness of the deposited Ge ~4 ML, three-dimensional islands begin to nucleate between the pit period of 0.7 µm or greater (Figure 4 and Figure 5). For a period of 1 µm or larger, the deposited amount of Ge is sufficient to form a group of 8–10 islands around the perimeter of the pits (Figure 5). It was found that the deposition of an epitaxial Si/Ge structure with a total thickness of ~150 nm does not lead to complete overgrowth of the pits if the pit period is 1 µm or less. These non-overgrown pits, arranged in a square lattice, presumably can form a two-dimensional photonic crystal, the modes of which spectrally overlap with the luminescence signal of ordered Ge(Si) QDs. The PL studies of Ge(Si) QDs obtained on a pre-patterned surface of SOI substrate with different pit periods are presented below and confirm this assumption.

### 3.3. Luminescent Properties of the Structures with Ordered Ge(Si) QDs

Studies of the luminescent properties of structures with ordered Ge(Si) QDs are performed by the micro-PL method at 77 K with a 10× objective lens. This lens with a numerical aperture of NA = 0.26 allowed focusing the laser excitation beam with λ = 532 nm into a spot with a diameter of ~10 µm. The same lens provides the collection of the PL signal is within the solid angle 30° near the normal to the sample surface. That is, the signal was collected from the vicinity of the Г-point of the dispersion relation for the PhC formed by pits on the pre-patterned surface of SOI substrate.

**Figure 7:**
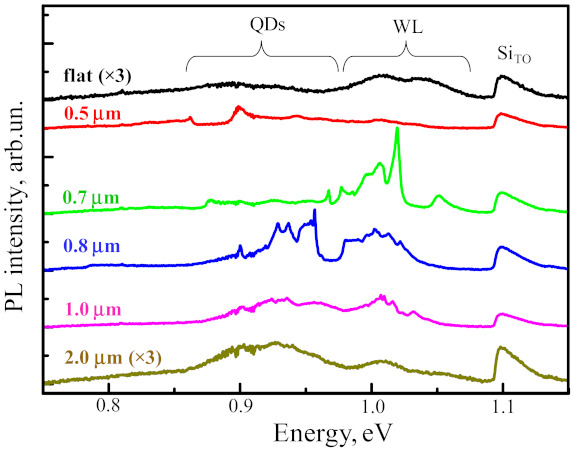
Micro-PL spectra measured in the non-patterned, flat area of structure (black) and pre-patterned areas with pit periods of 0.5 (red), 0.7 (green), 0.8 (blue), 1.0 (magenta) and 2.0 (dark yellow) µm. The SEM and AFM images of these areas are shown in Figure 4, Figure 5 and Figure 6. The pit periods are indicated near the corresponding spectra. The signals related to the transverse optical phonon-assisted signal of the Si layer (Si_TO_), the wetting layer (WL), and the QDs are indicated. The spectra from the non-patterned area and the area with a period of pits of 2 µm are multiplied by 3 for clarity. All the spectra were measured at 77 K.

In the micro-PL spectrum corresponding to the non-structured part of the sample, apart from the signal from Si, the signal related with the wetting layer in the energy range of 0.95–1.09 eV and a weak signal from QDs at lower energies were observed (Figure 7). The appearance of weak signal from QDs in the micro-PL spectrum of the unstructured area is due to the fact that the amount of deposited Ge (~4 ML) is enough only for the nucleation of QDs with low surface density on a flat Si surface. It should be noted that high growth temperature (700 °C) and low Ge growth rate (0.05 Å/s), which are facilitated to the spatial ordering of QDs, at the same time lead to the increase of Si content in Ge(Si) QDs [8,37]. The high Si content in Ge(Si) QDs is caused by the strain-driven alloying [38]. As a result of high Si content, the PL signal from ordered Ge(Si) QDs is obtained at higher energies, close to the signal from the wetting layer [8,39] (Figure 7).

**Figure 8:**
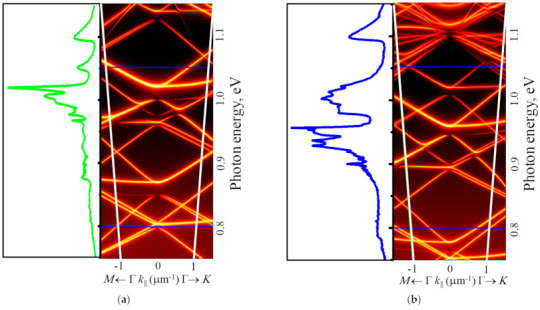
The emissivity near the Г-point simulated for the structured sample with the periods of PhC lattice a = 0.7 µm (**a**) and 0.8 µm (**b**) and the experimental micro-PL spectra from corresponding areas. For these periods and the pits radii r ~ 55 nm, the parameter r/a is equal to ~0.08 and 0.07 for figures (**a**) and (**b**), respectively. The emissivity is represented in the logarithmic color scale. White lines in the figures denote the 15° light cone from which the PL signal is collected. Horizontal blue lines bound the spectral range of intrinsic photoluminescence of Ge(Si) QDs and wetting layer.

The micro-PL spectra measured in areas with pits demonstrate their strong dependence on the pit period (Figure 7). To explain the influence of the PhC period (*a*) on the micro-PL spectra, it is necessary to account for (i) the experimental dependence of nucleation and growth processes of Ge(Si) QDs on the parameter *a*, which is described in the previous section, (ii) the close correlation between the spectral position and mode properties of the photonic crystal. To determine the dependence of PhC properties on the period, their optical states were simulated using the Fourier modal method in the scattering matrix form [28]. For simulations, the average values of the structure thickness above the buried oxide layer (~260 nm) and the pits radii (*r*) (*r*~55 nm) determined from the SEM images (Figure 6) were taken. By inspecting the SEM and AFM images (Figure 4 and Figure 5), one can assume that the average diameter of pits only weakly depends on the period. In this case, an increase in the pit period leads to the decrease of the *r/a* value. In simulations of PhCs emissivity, we assumed that Ge(Si) QDs are uniformly distributed within the whole structure. Such an assumption simplifies the simulations and does not affect the dispersion curves. The results of simulations of PhCs emissivities near the Г-point obtained for different periods together with the experimentally measured micro-PL spectra from corresponding areas are shown in Figure 8.

In the calculated dependences of PhCs emissivity, the observed maxima of the emissivity as a function of photon energy correspond to the quasiguided modes, which originate from the slab’s guided modes falling inside the light cone due to the structure periodicity (Figure 8). By analyzing the dispersion curves of the quasiguided modes, one can distinguish two types of modes differing in their emissivity in the Г-point. The modes of the first type are the optically bright, which are coupled with the far-field, thus having a low Q-factor due to the radiation losses [28,40,41]. In contrast, other modes in the Г-point are dark due to the symmetry mismatch with the propagating modes of the free space. These modes are often referred to as bound states in the continuum (BIC) modes. Due to weak coupling with the far-field near the Г-point, such modes are characterized by high values of the Q-factor [18].

A joint analysis of the simulated radiative properties of PhC (Figure 8) and the SEMand AFM images obtained for the areas with different pit periods (Figure 4, Figure 5 and Figure 6) allows us to explain the features of the observed micro-PL spectra (Figure 7 and Figure 8). In the micro-PL spectrum measured in an area with a pits period of 0.5 µm, there is only a small increase in the intensity of the luminescence signal in the energy range of 0.85–0.9 eV in comparison with the PL signal from the non-structured sample area (Figure 7). This increase is associated with the interaction of the QDs radiation with PhC modes, which, according to calculations, are located in this range of energy. The low intensity of the PL signal in this area of the structured surface is caused by the fact that for a period of 0.5 µm, the intensity of an initial PL signal in 0.85–0.9 eV energy range is small. This is due to the absence of three-dimensional Ge(Si) islands in this area, which could emit at 0.85–0.9 eV. According to the simulations, the other mode of a photonic crystal formed by pits located at a distance of 0.5 µm from each other, falling into the energy range of ~0.97 eV. In this energy range, only a weak PL signal is also observed in the initial structure, corresponding to an intermediate response between the photoluminescence signals of the wetting layer and Ge(Si) QDs (Figure 7).

At the same time, a significant (more than an order of magnitude) increase of the maximum intensity is observed in micro-PL spectra measured from the areas with the pits periods of 0.7 and 0.8 µm (Figure 7). The integrated intensity of the PL signal in these areas increased by 4–5 times with respect to the signal measured in the non-structured part of the sample. The main increase of the PL intensity in the areas with these periods of the location of the pits is observed in different spectral ranges corresponding to the signals associated with the wetting layer for a = 0.7 µm and with the Ge(Si) QDs for a = 0.8 µm (Figure 7). In the area with the pits period of a = 0.7 µm, the increase of PL intensity of the wetting layer alone is caused by the two factors. First, according to the results of SEM studies, the surface density of Ge(Si) QDs is still quite small for this period (Figure 4b). Second, according to the calculation data (see Figure 8a), in the spectral range of the wetting layer at ~1.02 eV, there is a high-emissivity mode of the PhC that can interact with an active medium.

An increase in the period of pits location to a = 0.8 µm leads to a number of significant changes. First, this leads to a shift of the PhC modes to the lower energy range. As a result, the PhC mode, which is characterized by a high emissivity, turns out to be consistent with the luminescent response of Ge(Si) nanoislands (in the energy range of ~0.95 eV, Figure 8b). Second, when the pits period increases from 0.7 to 0.8 µm, the Ge(Si) QDs surface density increases significantly (Figure 4). These two factors result in a significant increase of the G(Si) QDs related PL signal in the area with pits period a = 0.8 µm due to interaction of their emission with one of the high emissivity PhC modes (Figure 7 and Figure 8). With an increase in the period of the location of the pit at a fixed diameter, there is also an increase in the spectral density of the PhC modes, which is a consequence of a decrease in the r/a parameter (Figure 8). As a result, several modes with a sufficiently high emissivity occur within the spectral range of luminescence response of the structure, and, for the pits period of 0.8 µm, this gives rise to an increase of the PL signal associated not only with the Ge(Si) islands but also with the wetting layer and Si layers in the structure (Figure 7 and Figure 8).

As it has been shown above, an increase of the PhC period from 0.8 to 1 µm does not lead to a significant increase of the local (near the pits) and the total surface density of Ge(Si) QDs (see Figure 4c and Figure 5 for comparison). However, such a variation of the PhC period leads to significant changes of the PhC properties, namely, a number of the radiative modes, which are characterized by their relatively low peak emissivity within the spectral range of the luminescence responses related to the wetting layer and Ge(Si) QDs. As a consequence, an almost uniform increase of the signal intensity is observed in the micro-PL spectrum of the area with the PhC period of 1 µm in comparison with the non-structured sample area (Figure 7). In this case, an increase of the maximum and integrated PL intensity is significantly smaller than in the case of PhC period of 0.8 µm (Figure 7).

The PL intensity, which is observed in the structured sample area in comparison with the non-structured one, gradually decreases with a further increase of the pits period from 1 to 2 µm (Figure 7). This is due to the above-mentioned phenomenon of the increase of the overgrown pits fraction, with an increase of the distance between them (Figure 5). As a result, for large pits periods, the grown structure does not behave as a photonic crystal. In this case, the main reason for the higher intensity of the PL signal from Ge(Si) QDs from the region with a pit period of 2 µm as compared to the non-structured region is the higher surface density of QDs in structured regions with a pit period of 0.8 µm or larger.

It should be noted that the demonstrated increase of the PL intensity of the spatially ordered Ge(Si) QDs due to the interaction of their emission with the PhC modes, is obtained at low (77 K) temperature. At the same time, for the disordered Ge(Si) QDs, a significant increase of their PL intensity due to the interaction with radiative modes of the PhC has been observed at room temperature as well [41,42]. This fact could be explained by the high growth temperatures and low Ge deposition rates used for fabrication of the spatially ordered QDs, which, as was mentioned above, lead to the increase of the Si fraction in Ge(Si) QDs and, as a consequence, to the decrease of their PL intensity at room temperature [43]. Thus, used growth conditions are not optimal in terms of maximizing the PL signal from the Ge(Si) QDs at room temperature. As a result, to achieve a sufficient PL signal from them at room temperature, one has to modify the fabrication conditions of the ordered Ge(Si) QDs.

It is believed that the approach proposed in the present work can be used for the development of light sources for Si-based photonics, which fabrication process is compatible with modern CMOS technology. Electrical pumping of the PhCs with QDs, which is necessary for these purposes, can be realized, for example, by the formation of lateral p-i-n diodes on the grown structures [44,45].

## 4. Conclusions

Using Monte-Carlo simulation of Ge growth on a pit-patterned Si(001) surface, the optimal parameters of the array of pits (their shape, depth, and period), at which the nucleation of Ge(Si) QDs occurs not inside the pits but around them, or at the maximum distance from them were found. The simulation results were used to develop a new approach to the one-stage formation of a spatially ordered array of Ge(Si) QDs embedded into a two-dimensional photonic crystal. This approach consists of carrying out the heteroepitaxial growth of Ge(Si) QDs on a pre-patterned SOI substrate with deep pits with a sidewall inclination angle > 54°. It is shown that for certain parameters of the pit array, the growth of an epitaxial structure with QDs does not lead to overgrowth of pits located in a square lattice with a period of 0.5–1 µm, which guarantees the formation of a photonic crystal. An increase in the intensity of the luminescence signal from the wetting layer and Ge(Si) QDs at certain pit periods was observed, which is presumably associated with the interaction of the active medium with the radiation modes of the photonic crystal. This assumption is confirmed by numerical simulation of the optical properties of photonic crystals formed by the periodical arrays of uncovered pits located on the SOI substrate. The approach proposed in this work can be successfully used for embedding the spatially ordered QDs into different dielectric resonators.

## Supplementary Materials

The following are available online at https://www.mdpi.com/article/10.3390/nano11040909/s1, Figure S1: Calculated elastic energy distribution in the Ge layers deposited on pit-patterned Si(001) substrate.
