# Supplementary material for: One-Stage Formation of Two-Dimensional Photonic Crystal and Spatially Ordered Arrays of Self-Assembled Ge(Si) Nanoislandson Pit-Patterned Silicon-On-Insulator Substrate
Source: Nanomaterials (Basel). 2021 Apr 2;11(4):909. doi: 10.3390/nano11040909 (PMC8065389; doi:10.3390/nano11040909)
Supplement: Supplementary file 1 [file nanomaterials-11-00909-s001.pdf]

Supplementary Materials

One-Stage Formation of Two-Dimensional Photonic Crystal and Spatially Ordered Arrays of Self-Assembled Ge(Si) Nanoislands on Pit-Patterned Silicon-on-Insulator Substrate

The dependence of the QD nucleation sites on the pits shape was confirmed experimentally in [1], where the details of MC model calculations are also presented. This effect is a consequence of the fact that the elastic strain distribution at the Ge/Si interface depends on the shape of the pit bottom (Figure S1). According to the calculations the most relaxed region for a V-shaped pit is located in the center of the pit, where the nucleation of island occurs (Figure S1a,c). In the case of a pit with a U-shaped profile at the initial stages of deposition (less than 3 ML of deposited Ge), the most relaxed regions are located at the boundary between the flat bottom and the pit walls. During further Ge deposition, these regions are displaced upward along the pit walls towards its outer boundaries. As a result, at 4.3 ML of Ge deposited, the most relaxed areas are found at the top pit edges, where 3D islands nucleate (Figure S1 b,d).

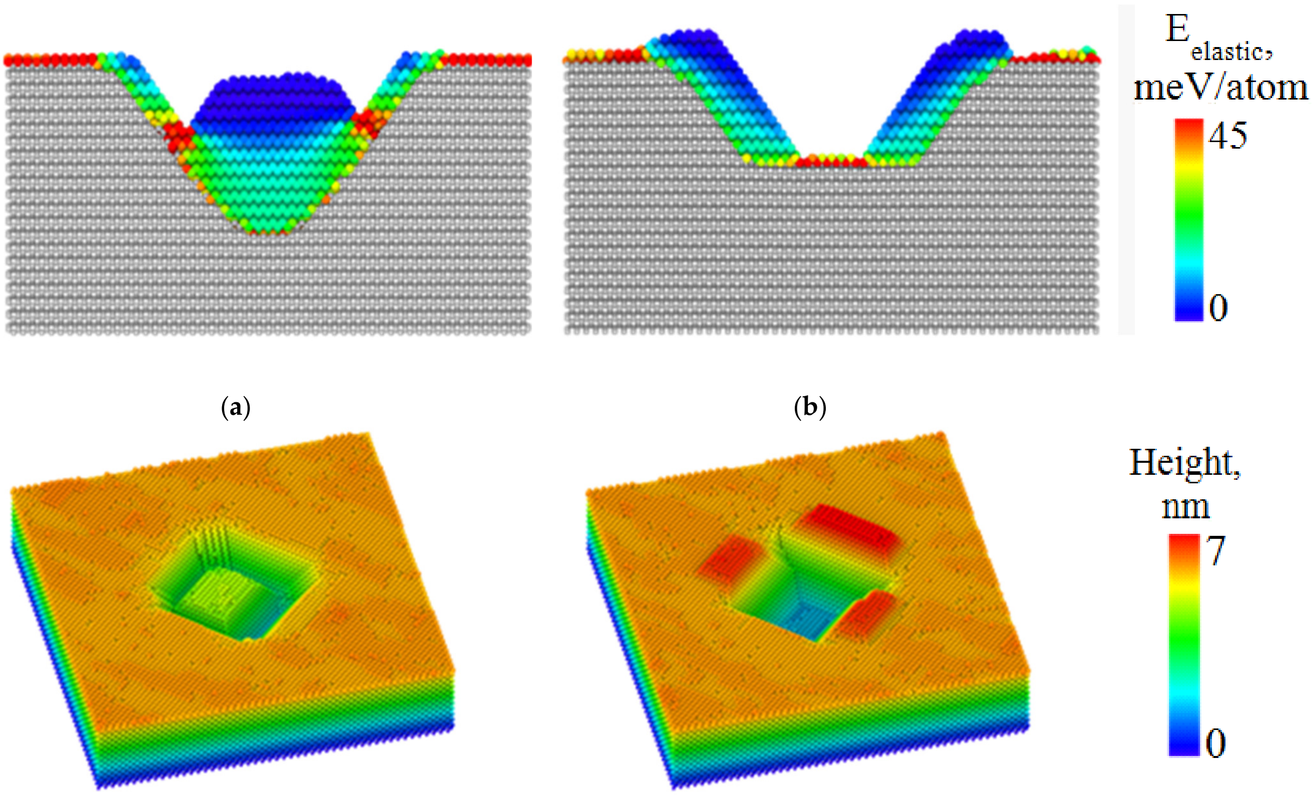

(**a**) (**b**)

(**c**) (**d**) (**b**)

Figure S1. (**a**,**b**): Calculated elastic energy distribution in the Ge layers deposited on pit-patterned Si(001) substrate with (**a**) V- and (**b**) U-shaped pits. The amount of Ge deposited is 4.3 ML. The rate of Ge deposition is 0.1 ML/s. The growth temperature is 450 °C. The elastic energy magnitude per Ge atom is indicated by the color legend. Si atoms are colored gray. The pit sidewall inclination angle is 54°. (**c**), (**d**): The images of QDs formed during Ge deposition on the patterned Si surface with (**c**) V- and (**d**) U-shaped pits.

[1] Smagina, Zh.V.; Zinovyev, V.A.; Rudin, S.A.; Novikov, P.L.; Rodyakina, E.E.; Dvurechenskii, A.V. Nucleation sites of Genanoislands grown on pit-patterned Si substrate prepared by electron-beam lithography. *J. of Appl. Phys.*2018, *123*, 165302 (5) (DOI: 10.1063/1.5009154).
